# Detection of Pyrethroids in Food by Immunofluorescence Enhanced Method Based on Three-Layer Core-Shell Structure Upconversion Materials

**DOI:** 10.3390/foods11070990

**Published:** 2022-03-29

**Authors:** Lingyan Zhao, Jingyi Jin, Wenbo Zhu, Yuehua Zuo, Yang Song

**Affiliations:** 1 Tianjin Key Laboratory of Animal and Plant Resistance, Tianjin Normal University, Tianjin 300387, China; 2010170003@stu.tjnu.edu.cn (L.Z.); 2110170005@stu.tjnu.edu.cn (J.J.); 1910170006@stu.tjnu.edu.cn (W.Z.); 2 Technical Center for Safety of Industrial Products of Tianjin Customs District, Tianjin 300308, China; zuoyuehua1987@126.com; 3 State Key Laboratory of Food Nutrition and Safety, Tianjin University of Science and Technology, Tianjin 300457, China

**Keywords:** pyrethroids, upconversion nanomaterials, fluorescence immunoassay

## Abstract

A novel rare earth upconversion nanomaterial with a three-layer sandwich core–shell structure was synthesized by an improved thermal decomposition method, and the morphology, fluorescence intensity and diffraction peak position of the new material were characterized by TEM (transmission electron microscope), XRD (Powder X-ray diffraction)and fluorescence spectrophotometer. The inert core/active shell/inert shell design improved the upconversion luminous efficiency of the new material several times. FTIR (Fourier transform infrared spectroscopy)characterization showed that the surface of activated upconversion nanoparticles was modified with silicon shell and amino group. Combined with the characteristics that aminoated polystyrene magnetic microspheres could be separated by the magnetic field, an upconversion magnetic separation immunoassay method for the detection of pyrethroid pesticide residues was established. The capture probe competed with the pyrethroid standard, combined the signal probe, and measured the fluorescence signal value formed by the capture probe signal probe complex at 542 nm under 980 nm excitation light. The LOD (limit of detection)of fenpropathrin was 0.01 μg/L, cypermethrin was 0.015 μg/L, and fenvalerate was 0.011 μg/L. Through the actual sample detection of apple, cabbage and other samples, the recovery rate of pyrethroids was between 83.4~97.8%. The comparison with the HPLC (high performance liquid chromatography)detection results showed that the established method had good accuracy, and could realize the quantitative analysis of pyrethroids in food.

## 1. Introduction

Upconversion nanomaterials (UCNPs) follow the anti-stoke luminescence law, which could convert long wave low-energy photons into short wave high-energy photons [1,2], and have stable photochemical properties. Rare earth element doped upconversion materials could not only emit strong visible light under the excitation of near-infrared but also have the advantages of small light damage, high penetration depth, low spontaneous fluorescence interference of biological tissue and low biological toxicity [3,4,5,6]. Therefore, they have attracted extensive attention in the fields of medical imaging, detection and sensing. UCNPs could be combined with fiber chromatography materials to prepare test strips to detect veterinary drug residues in aquatic products and livestock and poultry meat. They could also be used to develop quantum dot biosensors to detect heavy metal ions in food, which shows that UCNPs have broad application prospects in food safety detection. However, the defects of low luminous efficiency and short fluorescence life have limited the research and application of UCNPs. Therefore, researchers at home and abroad have prepared upconversion materials with core–shell structures to improve their optical properties. For example, in 2020, Wu Haoyu prepared inert core–shell structure NaYF_4_:Yb/Er@NaGdF_4_, and compared with NaYF_4_:Yb/Er without shell, its luminous efficiency was increased by more than 20 times [7]. In the same year, Zhu Yuyan prepared the active core–shell structure NaYbF_4_@NaYF_4_:Yb/Er, and the surface was coated with NaYbF_4_ shell doped with sensitizer Yb^3+^; compared with shell-free nanoparticles NaYF_4_:Yb/Er, the fluorescence intensity was increased by 6 times [8]. In addition to the double-layer core–shell structure, Qiu et al. prepared the NaYF_4_:Yb^3+^/Tm^3+^@NaYbF_4_@/NaYF_4_ multilayer core–shell structure of active core/active shell/inert shell, which increased the fluorescence brightness by 11 times compared with NaYbF_4_@/NaYF_4_ [9]. The research and development of multi-layer core–shell structure had created space confinement, doped rare earth ions in different shells, improved the upconversion efficiency and reduced the occurrence of cross relaxation [10].

Pyrethroids have been mainly used for the control of vegetable and fruit pests in agricultural planting and have become the second most common insecticide in agricultural production, accounting for about 30% of the pesticide market [11,12,13,14]. The latest national standard GB 2763-2021 stipulates that the maximum residue limit of fenpropathrin in fruit is 5 mg/kg, that in cabbage iss 1 mg/kg and that in cucumber is 0.2 mg/kg. The maximum residue limit of cypermethrin in apple, pear and cabbage is 2 mg/kg, and the maximum residue limit of fenvalerate in apple, pear and cabbage is 1 mg/kg; EU standard EU441/2012 stipulates that the maximum residue of bifenthrin in tomato is 0.3 mg/kg, and the maximum residues of fenvalerate and beta cypermethrin in potato are 0.02 mg/kg and 0.01 mg/kg [15,16,17]. Even though there are limited requirements in the national standards, the excessive residue of pyrethroid pesticides in edible agricultural products has been banned repeatedly. According to the national sampling data, pyrethroid pesticides are often used in leek, celery, green pepper, cowpea, pear and citrus, and the problem of unqualified rate was more prominent [18,19,20].

In this experiment, three-layer sandwich structure rare earth UCNPs were synthesized by the classical thermal decomposition method. The silicon shell and amino group were modified on the surface of UCNPs by the Stober method to increase hydrophilicity and biocompatibility. Based on the principle of upconversion magnetic separation immunofluorescence, an immunoassay method for rapid detection of pyrethroid residues in food was proposed. The additional amount of antigen and antibody in the detection system was optimized through bicinchonininc acid (BCA)kit to determine the optimal reaction conditions; to realize the rapid determination of pyrethroids in apple, pear, cabbage and cucumber samples, the accuracy of the method was verified by HPLC comparison experiment.

## 2. Materials and Methods

### 2.1. Materials and Instruments

Fenpropathrin, cypermethrin and fenvalerate were purchased from Adamas company (Shanghai, China). Enzyme labeled secondary antibody (Sheep anti rabbit) was purchased from Shanghai TITAN Technology Co., Ltd. (Beijing, China). Pyrethroid universal antigen, pyrethroid universal antibody, YCl_3_·6H_2_O, YbCl_3_·6H_2_O, ErCl_3_·6H_2_O were purchased from Bioengineering Shanghai Co., Ltd. (Shanghai, China). 1-octadecene, ammonium fluoride and 3-aminopropyl triethoxysilane (APTES, 98%) were purchased from Tianjin Hengshan Chemical Technology Co., Ltd. (Tianjin, China). Sodium hydroxide, methanol, absolute ethanol, acetonitrile and sodium chloride were purchased from Tianjin Chemical Reagent Supply and marketing company (Tianjin, China). Oleic acid, tetraethoxysilane (TEOS, 98%), CO-520 and cyclohexane were purchased from Tianjin Baiaotai Technology Development Co., Ltd. (Tianjin, China). Bovine serum albumin (BSA, 98%) was purchased from Merck, Germany. Glutaraldehyde was purchased from Shanghai Meiruier Chemical Technology Co., Ltd. (Shanghai, China). The ammonia and BCA protein concentration determination kit was purchased from Tianjin Yuanli Chemical Co., Ltd. (Tianjin, China). Aminoated polystyrene magnetic microspheres (MPM) were purchased from Beijing Beisile Color Co., Ltd. (Tianjin, China). The nanoparticle morphology and size of UCNPs were determined by FEI TECNAI G20 transmission electron microscope (TEM, FEI Company, Hillsboro, OR, USA). Fourier transform infrared spectroscopy (FTIR) of UCNPs was determined by FTIR spectrophotometer (Perkin Elmer, Waltham, MA, USA). Powder X-ray diffraction (XRD) measurements of UCNPs were performed by the AXIS-ULTRA-DLD instrument (Millipore, New York, NY, USA). All the above instruments were from the school of the chemistry of Tianjin Normal University (Tianjin, China). The fluorescence intensity of upconversion nanoparticles was detected by an f-2500 fluorescence spectrophotometer equipped with a 980 nm laser exciter. Waters e2695 HPLC was from Thermo Fisher Scientific, Waltham, MA, USA. All chemicals used were analytical grade.

### 2.2. Preparation and Surface Modification of UCNPs

Based on the high-temperature thermal decomposition method, 2 mmol YCl_3_·6H_2_O, 30 mL1-octadecene and 12 mL oleic acid were put into a three-mouth bottle and heated to 170 °C under high-purity argon to form a yellow transparent solution. When the reactant was cooled to room temperature, methanol solution dissolved with 5 mmol NaOH and 8 mmol NH_4_F was added drop by drop, stored at room temperature for 30 min, slowly raised the temperature to 100 °C, and methanol and water was evaporated in the reaction system. Following this, the temperature was raised again to 300 °C and kept for 1.5 h. After the reaction system was cooled to room temperature, the sample was centrifuged at 8500 rpm, washed several times alternately with ethanol and cyclohexane, dried in the oven and the NaYF_4_ core was collected.

In additional sample preparation, 2 mmol RECl_3_·6H_2_O (RE:78% mol Y^3+^, 2% mol Er^3+^, 20% mol Yb^3+^), 12 mL oleic acid and 30 mL octadecene was placed into a three-mouth bottle, raised the temperature to 170 °C under argon to form a yellow transparent solution, and cooled to room temperature. Next was the addition of 2 mmol NaYF_4_ core dissolved in cyclohexane dropwise and stirred continuously, and the addition of methanol solution dissolved with NaOH and NH_4_F and stirred continuously at room temperature. The reactant was heated to 100 °C and cyclohexane was evaporated. After the removal of cyclohexane, the procedure was the same as above. After being cleaned several times, it was dried in the oven and collected NaYF_4_@NaYF_4_:Yb,Er.

In addition, the same amount of YCl_3_·6H_2_O, octadecene and oleic acid was placed into a three mouth bottle and heated to 170 °C to form a yellow transparent solution. This was then cooled to room temperature, to which was added 2 mmol NaYF_4_@NaYF_4_:Yb,Er dissolved in cyclohexane dropwise and stirred slowly. After cyclohexane removal, nucleation and growth, washing, drying and collection, the NaYF_4_@NaYF_4_:Yb, Er@NaYF_4_ nanoparticles with the three-layer sandwich structure of inert core/active shell/inert core were obtained.

Finally, 100 mg UCNPs was weighed out, dispersed in 50 mL ethanol by ultrasonication and stirred quickly. Following this, 4 mL ammonia and 30mL secondary water was added and underwent vigorous stirring at 45°C. Four microliter TEOS was added to the mixture within 5 h. And one hundred microliter APTES solution was added dropwise to the suspension and reacted for 5 h. The sample was washed twice with ethanol and centrifuged to obtain white solid precipitation. This was then placed in the oven and dried for 24 h, after which UCNPs@SiO_2_@NH_2_ was collected and stored in dark condition prior to testing

### 2.3. Preparation of Capture Probe and Signal Probe

The signal probe was prepared by the glutaraldehyde crosslinking method. Here, 10 mg UCNPs@SiO_2_@NH_2_ was dissolved in 5 mL PBS (10 mmol/L), ultrasonicated for 20 min, 1.25 mL of 25% glutaraldehyde solution and 100 mg NaBH_4_ was added drop by drop, shaken slowly for 1 h, centrifuged to remove the supernatant, washed with PBS for three times, dispersed the precipitate in 5 mL PBS, ultrasonicated for 10 min, added a certain amount of antibody, reacted for 6 h, and centrifuged to collect the supernatant and precipitate respectively. Then the precipitate was dissolved in 5 mL PBS (including 1% BSA), reacted for 6 h, and the precipitate was collected by centrifugation, dispersed in 5 mL PBS and stored at 4 °C to obtain the signal probe with the concentration of 2 mg/mL. When preparing the capture probe, the same amount of amino magnetic microspheres was dissolved in 5 mL PBS. The operation was the same as above. Following the addition of a certain amount of antigen, and centrifugation, the capture probe with a concentration of 2 mg/mL was obtained and stored at 4 °C.

In the process of preparing the two probes, the addition amount of pyrethroid antigen and antibody was optimized. Addition of 20 μg, 40 μg, 60 μg, 80 μg, 100 μg antigen and antibody respectively was done in preparing capture probes and signal probes. Calculation was done of the coupling amount through the BCA kit, and then the coupling rate was calculated according to the following formula to determine the optimal addition amount of antibody (antigen).

(1) $$\mathrm{coupling}\;\mathrm{rate}=\frac{\mathrm{Mc}}{\mathrm{Mt}}\times 100\%$$

In the formula, Mc is the coupling amount of antibody (antigen), and Mt is the total amount of antibody (antigen).

### 2.4. Optimization of Capture Probe Addition

To ensure that the capture probe and signal probe reacted fully, the addition amount of capture probe was optimized in this study. When the addition amount of signal probe was determined, addition of 40 μg, 60 μg, 80 μg, 100 μg, 120 μg, 140 μg capture probes respectively, and the fluorescence value of the signal probe capture probe complex was measured.

### 2.5. Establishment of UpConversion Magnetic Separation Immunofluorescence Method

The pyrethroid standard competed with the capture probe to bind the signal probe. The capture probe signal probe conjugate was separated by the external magnetic field, and the upconversion magnetic separation immunofluorescence method was established. The pyrethroid standard competed with the capture probe to bind the signal probe. The capture probe signal probe conjugate was separated by the external magnetic field, and the upconversion magnetic separation immunofluorescence method was established. The standard solution was taken of fenpropathrin, cypermethrin and fenvalerate in different concentrations and mixed with the capture probe, added the signal probe, reacted at 25 °C for 1 h, separated the capture probe signal probe complex with the external magnetic field, collected the precipitation, washed it with PBS for three times, dispersed the precipitation in 2 mL PBS, and measured the fluorescence value with an external 980 nm excitation light source fluorescence spectrophotometer. The upconversion immunofluorescence standard curve at different concentrations was established.

### 2.6. Sample Pretreatment

Apple, pear, cabbage and cucumber were selected as experimental samples and respectively smashed in a blender. Following this, 2 g apple and pear samples were placed into a centrifuge tube, 10 mL acetone-n-hexane (1:1) mixed solvent was added, and subjected to vortexed oscillation for 10 s and ultrasonic extraction for 30 min. Then, sample was centrifuged at 4000 r/min for 5 min, and the supernatant was passed through a 0.22 µm micro membrane and stored for the test.

Two grams of cabbage and cucumber samples were prepared and placed in a centrifuge tube, to which 4 mL 0.1% acetic acid acetonitrile solution was added, and subjected to vortexed oscillation for 5 min; following this, 1 g sodium chloride was added, and subjected to vortexed oscillation for 2 min, centrifuged for 2 min at 4500 r/min, and 2 mL of supernatant was extracted. Finally, the sample was spun steamed and dried, fixed to a volume of 1 mL with methanol, passed through 0.22 µm microfilm, and stored for testing.

### 2.7. HPLC Comparison Test

#### 2.7.1. Chromatographic Conditions

Chromatographic conditions were as follows: Chromatographic column: Bridge C18 (5 µm, 4.6 × 250 mm); Mobile phase: methanol–water (32:68); Flow rate: 1.0 mL/min; Column temperature: 30 °C; Injection volume: 20 µL; Detection wavelength: 205 nm.

#### 2.7.2. Extraction and Purification

Samples were taken according to Appendix A of GB 2763-2021 [21]. Stems were removed from apple, pear and cucumber, and the root was removed from cabbage as the sample determination part. The samples were chopped and mixed into homogenate, sealed and frozen at −20 °C. Samples were extracted with acetonitrile, salted out and centrifuged with sodium chloride; the supernatant was purified by solid-phase extraction column, eluted with acetonitrile toluene solution (3 + 1), spun in the water bath until it was nearly dry, volume was fixed methanol, and it was filtered through 0.22 µm filter membrane, in preparation for test [22].

### 2.8. Actual Sample Testing

Three samples of apple, pear, Chinese cabbage and cucumber were randomly selected in the market, and the surface attachments were gently wiped with gauze. No pyrethroid pesticides were detected in 12 samples by HPLC. Three standards of fenpropathrin, cypermethrin and fenvalerate were randomly added into 12 blank samples. After the sequence was disrupted, they were renumbered 1–12#, tested simultaneously by this experimental method and HPLC, and the data were recorded.

### 2.9. Method Specificity

Fenpropathrin, Cypermethrin, Fenvalerate and their structural analogs bifenthrin, deltamethrin, homeopathic permethrin and four other pesticides commonly used in agricultural production were selected for cross-reaction experiments. Ten pesticides were configured with the same concentration as the standard and competed with the capture probe for the signal probe. There were detected and recorded at a fluorescence value of 542 nm. The fluorescence intensity after the reaction of the capture probe and the signal probe was recorded as I_0_, the fluorescence intensity after the reaction of different standards with the capture probe and the signal probe was recorded as I, and the difference between I_0_ and I was calculated as ΔI.

## 3. Results and Discussion

### 3.1. Characterization of Upconversion Materials

Rare earth doped upconversion nanomaterials have excellent physical and chemical properties, so they have been widely used. However, the low luminous efficiency had become the biggest resistance to the research and development of upconversion nanoparticles. The design of core–shell structure could greatly make up for the defects of upconversion materials. Rare earth upconversion materials are generally composed of matrix, sensitizer and activator. The choice of matrix materials could be oxides, sulfides, halides, etc., but the high phonon energy of oxides was not conducive to the occurrence of upconversion, and the iodide and bromide in halides were easy to be decomposed by moisture, which was not conducive to the preparation of materials. Therefore, fluoride was selected as the matrix material. NaYF_4_ in fluoride had good stability and high emission efficiency and was the first choice of matrix materials. The rare earth ions doped with upconversion nanomaterials had low photon utilization in the near-infrared region. If the absorption efficiency was increased by increasing the concentration of doped ions, the distance between activator ions was too close, and cross-relaxation will occur, resulting in concentration quenching and the luminous efficiency could not be improved. To improve the luminescence efficiency, a higher proportion of sensitizer ions could be added to the upconversion nanocrystals. Compared with other sensitizer ions, Yb^3+^ had the widest absorption cross-section in the near-infrared region. The activator ion Er^3+^ not only had a rich energy level structure but also had a narrow luminescence band, which could have efficient energy transfer with the sensitizer ion Yb^3+^. Due to the surface of rare earth upconversion nanocrystalline NaYF_4_:Yb,Er@NaYF_4_ being in direct contact with solvent and environment, a large number of ions were able to reach the exciting state transfer energy to the surface of upconversion nanoparticles and return to the ground state in a nonradiative transition, resulting in surface quenching, which greatly affects the luminescence efficiency of upconversion. Therefore, to suppress the occurrence of surface quenching, a layer of inert material was usually coated on the outside. As an inert shell, NaYF_4_ could not only have better lattice matching with the template seed crystal and better coating effect, but also because NaYF_4_ is an inert material, it inhibits the nonradiative relaxation of excited Er^3+^, making the energy transfer between Er^3+^ for a longer time, and increased the fluorescence lifetime of the material. Therefore, inert core/active shell/inert core three-layer sandwich structure upconversion nanoparticles NaYF_4_@NaYF_4_:Yb, Er@NaYF_4_ were designed.

**Figure 1:**
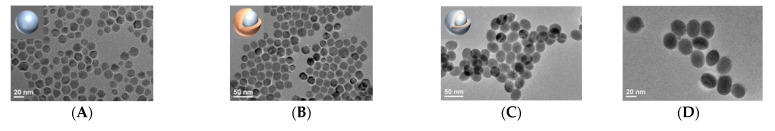

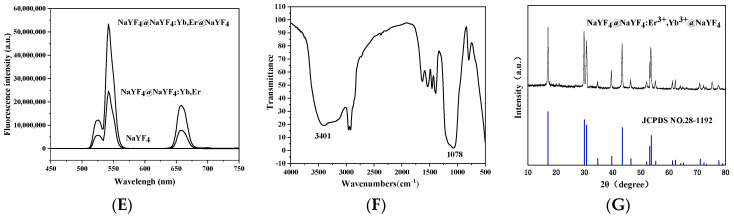
(**A**) TEM images of NaYF_4_; (**B**) TEM images of NaYF_4_:Yb,Er@NaYF_4_; (**C**) TEM images of NaYF_4_@NaYF_4_:Yb,Er@NaYF_4_; (**D**) TEM images of NaYF_4_@NaYF_4_:Yb,Er@NaYF_4_@SiO_2_@NH_2_; (**E**) Flu-orescence spectra; (**F**) FTIR spectra of NaYF_4_@NaYF_4_:Yb,Er@NaYF_4_@SiO_2_@NH_2_; and (**G**) XRD pat-tern of NaYF_4_@NaYF_4_:Yb,Er@NaYF_4_.

The morphology and size of upconversion nanomaterials were measured by transmission electron microscope. As shown in Figure 1, upconversion particles had good dispersion, uniform size and hexagonal phase. Figure 1A showed the inert core NaYF_4_ with a particle size of about 16 nm, Figure 1B showed the morphology of the inert core NaYF_4_ coated with the active shell, NaYF_4_:Yb, Er@NaYF_4_ about 22 nm, Figure 1C showed the morphology of NaYF_4_: Yb, Er@NaYF_4_ coated with the inert shell, and NaYF_4_@NaYF_4_:Yb, Er@NaYF_4_ about 26 nm. The coating of the core–shell structure could be proved to be successful by increasing the particle size. The fluorescence spectrum of UCNPs was measured by using a fluorescence spectrophotometer with an external 980 nm excitation light source. It could be seen from Figure 1E that the nanoparticles had the largest characteristic emission peak at 542 nm. The fluorescence efficiency of the nanomaterial was significantly enhanced after coating the active shell, and the fluorescence intensity increased several times after coating the inert shell. Compared with the core–shell structure NaYF_4_:Yb,Er@NaYF_4_ synthesized by Nahid ghazyani [23], the fluorescence intensity increased nearly 100 times. The coating of the inert shell greatly shortened the distance between the luminous center and the energy receptor, shortened the path from the sensitizer to the activator, reduced the energy loss, had high quantum efficiency, and realized high-strength emission.

To increase the dispersion and biocompatibility of upconversion materials in the aqueous system, they were modified with a layer of SiO_2_ shell and amino group on the surface by the classical Stober method. Figure 1D shows the transmission electron microscope image of the modified upconversion material. It could be seen from the figure that a thin layer of silicon shell appears on the surface of the modified UCNPs, and the modified particle size was about 30 nm, which indicated that the silicon shell on the surface of UCNPs was coated successfully. The functional groups on the surface of the modified nanomaterial were characterized by Fourier infrared spectroscopy. As shown in Figure 1F, the siloxy symmetric stretching and stretching vibration of the nanoparticle appeared in the area of about 1100 cm^−1^, which again showed that the material had been coated with a silicon shell. The stretching and bending vibration of the amino group appeared at 3401 cm^−1^, indicating that the surface of the material had been modified with the amino group. The peak at 2900 cm^−1^ in the figure was methylene symmetrical stretching vibration, which may be due to accidental contamination with substances containing methylene during tablet pressing, which will not affect the experimental results. The results of X-ray diffraction of the nanomaterial are shown in Figure 1G, and the diffraction peak was consistent with the comparison result of hexagonal beta phase UCNPs standard spectrum (JCPDS No. 28-1192), which further showed that the synthesized UCNPs was a hexagonal phase. The phonon energy of the hexagonal phase was lower than that of the cubic phase, which was helpful to improve the upconversion luminous efficiency of the material and was more suitable to be used as an upconversion matrix material.

### 3.2. Optimization of Antigen and Antibody Addition

Based on the principle of immunology, the signal probe was made by connecting pyrethroid universal antibody with modified upconversion particles by glutaraldehyde cross-linking method, and the capture probe was made by connecting pyrethroid universal antigen with amino magnetic microspheres.

**Figure 2:**
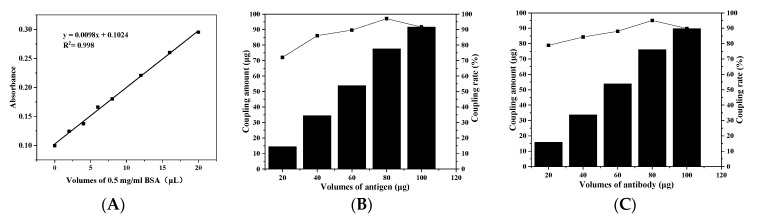
(**A**) BCA kit standard curve; (**B**) Optimization of the added amount of pyrethroid universal antigen conjugated with MPMs; and (**C**) Optimization of the added amount of pyrethroid universal antibody conjugated with UCNPs.

Figure 2 shows the detection results of antigen–antibody concentration in the supernatant after universal antigen and antibody were combined with magnetic particles and upconversion materials respectively. As shown in Figure 2B, when the addition amount of pyrethroid universal antigen was 20~80 μg, the coupling rate increases with the increase of the additional amount. When the addition amount was 80 μg, the coupling rate between antigen and amino magnetic microspheres reached the maximum. When the addition amount was 80~100 μg, the coupling rate decreased gradually. So when 80 μg antigen was added, the antigen combined with the amino magnetic microspheres performed best, and the coupling rate was 97.01%. Figure 2C shows that when the addition amount of pyrethroid universal antibody was 20~80 μg, the coupling rate increased gradually. When the amount of antibody added was 80 μg, the coupling rate between antibody and upconversion material reached the maximum. When the addition amount was 80~100 μg, the coupling rate decreased gradually. Therefore, 80 μg antibody was added when preparing the signal probe, and the coupling rate was 95.06%.

### 3.3. Optimization of Capture Probe

In the upconversion magnetic separation detection system, the capture probe was used as the antigen carrier and separation medium. If the addition amount was insufficient, it will affect the full binding of antigen and antibody, and finally lead to the weak fluorescence signal, so the fluorescence spectrophotometer could not detect the change of fluorescence signal difference. If the addition was excessive, the color of the reaction system will be darker, which may cause background interference, affect the fluorescence value and lead to waste.

**Figure 3:**
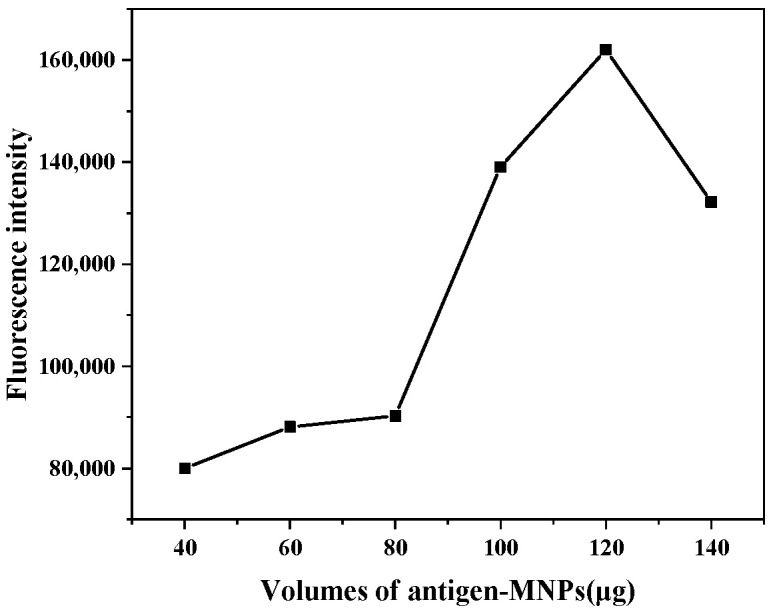
Optimization of capture probe addition.

On the premise of determining the additional amount of signal probe, the addition amount of capture probe was optimized. The optimization results of the addition amount of capture probe are shown in Figure 3. When the addition amount of capture probe was 40~120 μg, the fluorescence value of the capture probe signal probe complex gradually increased. When the addition amount of the capture probe was 120 μg, the fluorescence value of the reaction system reached the maximum. When the addition amount of capture probe was 120~140 μg, the fluorescence value of the capture probe signal probe complex decreased gradually. To achieve the best detection results without wasting materials, the optimal addition amount of capture probe was 120 μg.

### 3.4. Drawing of Standard Curve and Determination of Detection Limit

**Figure 4:**
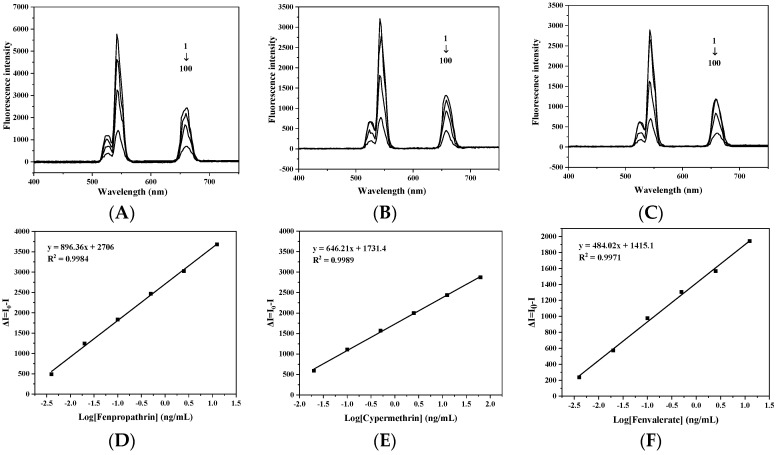
(**A**) Upconversion fluorescence spectrum of fenpropathrin at different concentrations; (**B**) Upconversion fluorescence spectrum of cypermethrin at different concentrations; (**C**) Upconversion fluorescence spectrum of fenvalerate at different concentrations; (**D**) Standard curve of fenpropathrin; (**E**) Standard curve of cypermethrin; and (**F**) Standard curve of fenvalerate.

For this procedure, 50 µL standard solutions of fenpropathrin, cypermethrin and fenvalerate of different concentrations and 120 µL capture probe were prepared, placed into the centrifuge tubes, to which 200 µL signal probe was added, and subjected to competitive reaction for 60 min. The signal probe capture probe conjugate was collected by the magnet and washed 3 times with PBS. The precipitate was dispersed in 2 mL PBS again, and the fluorescence intensity at 542 nm was measured by a fluorescence spectrophotometer with an external 980 nm laser. The logarithm was taken of the concentrations of fenpropathrin, cypermethrin and fenvalerate as the abscissa and the fluorescence intensity difference (∆I = I_0_ − I) of immune complexes in the detection system containing different concentrations of standards as the ordinate, and the standard curve for the detection of fenpropathrin, cypermethrin and fenvalerate was established. The fluorescence spectra at different concentrations of fenpropathrin, cypermethrin and fenvalerate are shown in Figure 4. With the increase of standard concentration, the fluorescence intensity in the reaction system decreased. According to the standard curve, it could be found that the LOD and sensitivity of upconversion magnetic separation immunoassay for fenpropathrin were 0.01 μg/L and 1.6 μg/L, for cypermethrin were 0.015 μg/L and 1.3 μg/L, and for fenvalerate were 0.011 μg/L and 1.5 μg/L. Liu Mengli [24] constructed a label-free fluorescence analysis method using NaYF_4_:Yb,Er. The LOD of fenpropathrin in traditional Chinese medicine was 0.24 ng/mL, the fluorescence intensity of NaYF_4_@NaYF_4_:Yb,Er@NaYF_4_ synthesized by this method was higher, and the LOD of upconversion magnetic separation immunoassay for pyrethroids was lower. This immunoassay method was more convenient, sensitive and efficient than most traditional methods for the detection of pyrethroids. The research of this method provides a powerful means for the detection of pyrethroids in food.

### 3.5. Elimination of Matrix Effect

**Figure 5:**
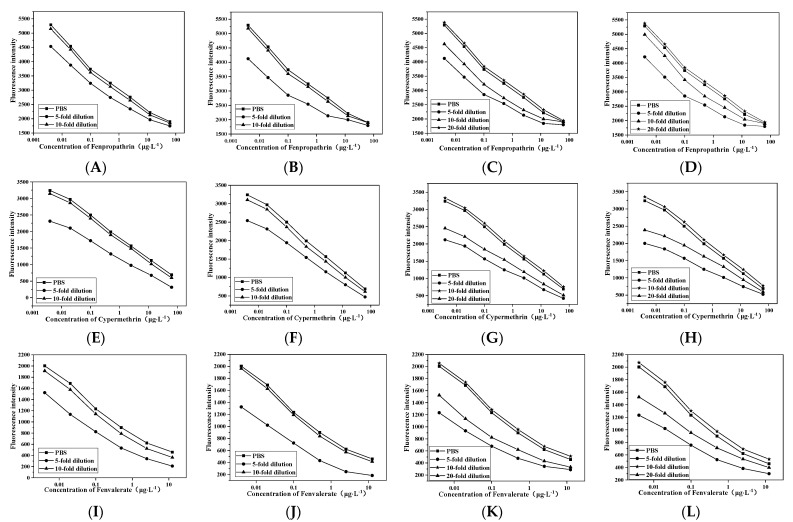
(**A**,**E**,**I**) Elimination results of matrix effect of cucumber; (**B**,**F**,**J**) cabbage; (**C**,**G**,**K**) apple; and (**D**,**H**,**L**) pear.

Proteins, sugars and pigments in the sample will interfere with the test results. The influence of the matrix could be reduced by sample dilution. Apple, pear, cabbage and cucumber samples were extracted with acetonitrile and diluted with PBS 5, 10 and 20 times respectively. The standard inhibition curve was drawn with the concentration of pyrethroids as the abscissa and the fluorescence intensity as the ordinate. The results are shown in Figure 5. When cucumber and cabbage samples were diluted 10 times and apple and pear were diluted 20 times, the standard inhibition curve was similar to the standard curve of the established method. It showed that the matrix effect could be eliminated when the cabbage and cucumber were diluted 10 times, and apple and pear were diluted 20 times.

### 3.6. Add Sample Recovery

All samples were detected by HPLC without pyrethroids before the addition and recovery experiment. Pyrethroid standards with low, medium and high concentrations were added to cucumber, cabbage, apple and pear respectively, and five parallel groups were set for the determination of each concentration of each sample. The same amount of standard with the same concentration was added to the same five samples, and the concentration of standard was determined by the method established in this paper. After sample pretreatment and PBS dilution, they were used for sample determination. Calculation of the recovery and CV of spiked samples was made according to the formula. As shown in Table 1, the average recovery of fenpropathrin was 88.6~97.8% and CV was 2.1~7.1%; the average recovery of cypermethrin was 84.5~93.8% and CV was 2.1~7.4%; the average recovery of fenvalerate was 83.4~94.2% and CV was 1.9~6.8%. The CV was not higher than 7.4%, indicating that the method established in this paper had good precision. The detection limit of apple and pear samples was 1 μg·kg^−1^, the detection limit of cabbage and cucumber was 0.5 μg·kg^−1^; it was far lower than the national standard of MRL in fruits and vegetables [21]. It showed that the method has good accuracy and could be used for the detection of pyrethroids in daily life.

(2) $$\mathrm{rate}\;\mathrm{of}\;\mathrm{recovery}\%=\frac{\mathrm{Detection}\;\mathrm{amount}\;\mathrm{of}\;\mathrm{spiked}\;\mathrm{sample}}{\mathrm{Standard}\;\mathrm{addition}}\times 100\%$$

(3) $$\mathrm{CV}=\frac{\mathrm{SD}}{\mathrm{MN}}\times 100\%$$

In the formula, CV is the coefficient of variation, SD is the standard deviation, and MN is the average value.

### 3.7. Comparison Experiment of High-Performance Liquid Chromatography

**Figure 6:**
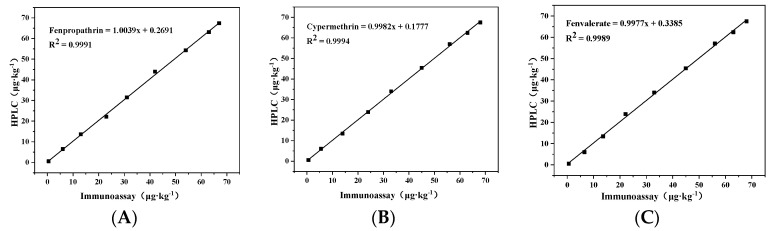
Correlation between HPLC and this research method in the detection of (**A**) fenpropathrin; (**B**) cypermethrin and (**C**) fenvalerate.

To verify the accuracy of upconversion magnetic separation immunoassay, the concentration of pyrethroids in blind samples was determined by HPLC. One sample was divided into two parts, and the same amount of standard was added respectively. One part was pretreated according to the description in part 2.6, and the method established in this paper was used for detection; another part of the samples was pretreated as described in Section 2.7 and detected by HPLC. Observation of the correlation between the results of the two detection methods was made. The retention time of blank spiked samples was 16.5 min for fenpropathrin, 18.7 min for cypermethrin and 21.3 min for fenvalerate. The correlation between the HPLC method and this research method is shown in Figure 6; the R^2^ of the correlation equation was greater than 0.995, indicating that the two methods had a good consistency. It showed that the method established in this paper is accurate and reliable. The upconversion magnetic separation immunofluorescence assay was accurate, and could be used for the detection of pyrethroids in real samples.

### 3.8. Actual Sample Test Results

The prepared blind samples were tested by this experimental method. The results are shown in Table 2. Three positive samples were detected in three cucumber blind samples; among the three blind samples of cabbage, two positive samples were detected, and the rest were negative samples; two positive samples were detected in three blind apple samples, and the rest were negative samples; three positive samples were detected in three blind pear samples. The results obtained by HPLC and this experimental method were compared, and the number of positive samples detected by the two methods was the same. The accuracy and practical applicability of the method established in this paper were evaluated by adding the recovery experiment and the actual sample detection experiment. The results showed that the upconversion magnetic separation immunofluorescence assay established in this experiment had good accuracy and could be applied to the detection of pyrethroid pesticide residues in vegetables.

### 3.9. Method Specificity Evaluation

**Figure 7:**
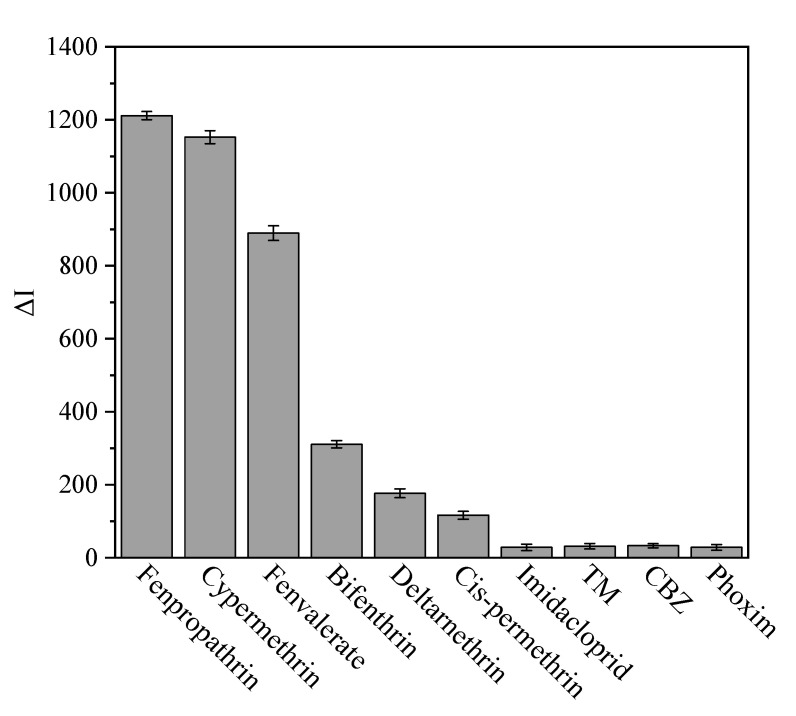
Cross reaction.

As shown in Figure 7, when the added standard was fenpropathrin, cypermethrin and fenvalerate, the change range of fluorescence intensity was large, indicating that the signal probe was specifically combined with fenpropathrin, cypermethrin and fenvalerate. When the added standard was its structural analogs bifenthrin, deltamethrin and cis-permethrin, the change range of fluorescence intensity was relatively large, indicating that the signal probe can also be combined with other pyrethroid standards, this method was suitable for the detection of pyrethroids. When the standard was other pesticides, the change range of fluorescence intensity was small, the signal probe had almost no specific binding with the standard, and there was only some nonspecific adsorption.

## 4. Conclusions

In this study, a new three-layer core-shell structure upconversion nanoparticle was synthesized. The activated material was coupled with pyrethroid antibody to prepare a signal probe, and the amino polystyrene magnetic microspheres were coupled with pyrethroid antigen to prepare a capture probe. Based on the specific binding of antigen and antibody and the characteristics that the magnetic microspheres could be separated by the external magnetic field, an upconversion magnetic separation immunofluorescence method for detecting pyrethroid residues was established. The upconversion magnetic separation immunofluorescence method for the detection of pyrethroid residues established in this paper had higher accuracy and lower detection limit than enzyme-linked immunosorbent assay; compared with the large-scale instrument detection method, this method has the advantages of being fast, convenient and low cost. The establishment of the upconversion magnetic separation immunofluorescence method enriches the technical means of the rapid detection market and provides theoretical support for the detection of pyrethroid residues.

## Figures and Tables

**Table 1:** Recovery rate of pyrethroids in added samples.

Sample	Additive Concentration (μg·kg^−1^)	Fenpropathrin			Cypermethrin			Fenvalerate		
		Mean ± SD	Average Recovery	CV	Mean ± SD	Average Recovery	CV	Mean ± SD	Average Recovery	CV
		(μg·kg^−1^)	(%)	(%)	(μg·kg^−1^)	(%)	(%)	(μg·kg^−1^)	(%)	(%)
Cucumber	0.5	0.44 ± 0.03	88.6	7.1	0.42 ± 0.03	84.5	7.4	0.42 ± 0.03	83.4	6.7
	2	1.83 ± 0.08	91.5	4.6	1.72 ± 0.07	86.2	4.1	1.71 ± 0.06	85.4	3.6
	10	9.31 ± 0.27	93.1	2.9	8.87 ± 0.19	88.7	2.1	8.79 ± 017	87.9	1.9
Cabbage	0.5	0.46 ± 0.03	92.7	6.1	0.45 ± 0.03	89.4	5.7	0.46 ± 0.03	92.6	5.8
	2	1.93 ± 0.09	96.5	4.5	1.83 ± 0.08	91.5	4.1	1.87 ± 0.07	93.4	3.9
	10	9.78 ± 0.36	97.8	3.7	9.33 ± 0.27	93.3	2.9	9.42 ± 0.23	94.2	2.4
Apple	0.5	0.44 ± 0.03	88.7	7.1	0.45 ± 0.03	89.6	6.9	0.45 ± 0.03	89.5	6.8
	2	1.83 ± 0.08	91.6	4.6	1.84 ± 0.07	92.1	3.9	1.81 ± 0.07	90.7	3.6
	10	9.51 ± 0.27	95.1	2.8	9.38 ± 0.27	93.8	2.9	9.29 ± 0.23	92.9	2.5
Pear	0.5	0.45 ± 0.03	89.7	5.9	0.44 ± 0.03	87.8	5.7	0.44 ± 0.03	88.7	5.8
	2	1.84 ± 0.08	91.9	4.2	1.78 ± 0.07	89.2	3.9	1.80 ± 0.07	89.9	4.1
	10	9.43 ± 0.20	94.3	2.1	9.18 ± 0.26	91.8	2.8	9.23 ± 0.22	92.3	2.4

**Table 2:** Detection of pyrethroids in actual samples.

	This Experimental Method			HPLC		
Sample	Fenpropathrin (mg·kg^−1^)	Cypermethrin (mg·kg^−1^)	Fenvalerate (mg·kg^−1^)	Fenpropathrn (mg·kg^−1^)	Cypermethrin (mg·kg^−1^)	Fenvalerate (mg·kg^−1^)
1#cucumber	-	-	85.6	-	-	86.4
2#cucumber	-	65.8	-	-	67.1	-
3#cucumber	57.3	-	-	58.6	-	-
4#cabbage	-	-	-	-	-	-
5#cabbage	78.2	-	-	79.5	-	-
6#cabbage	-	-	86.1	-	-	87.3
7#apple	-	-	46.1	-	-	47.6
8#apple	-	85.6	-	-	86.4	-
9#apple	-	-	-	-	-	-
10#pear	-	45.7	-	-	46.5	-
11#pear	-	-	66.5	-	-	67.9
12#pear	85.4	-	-	86.7	-	-

"-" indicates negative sample.

## Data Availability

Not applicable.
